# Inactivation of Single Strains of *Listeria monocytogenes* and *Salmonella* Typhimurium Planktonic Cells Biofilms With Plasma Activated Liquids

**DOI:** 10.3389/fmicb.2019.01539

**Published:** 2019-07-04

**Authors:** Cindy Smet, Marlies Govaert, Alina Kyrylenko, Md. Easdani, James L. Walsh, Jan F. Van Impe

**Affiliations:** ^1^Optimization in Engineering Center of Excellence, KU Leuven, Ghent, Belgium; ^2^CPMF^2^, Flemish Cluster Predictive Microbiology in Foods, Ghent, Belgium; ^3^BioTeC+ – Chemical and Biochemical Process Technology and Control, Department of Chemical Engineering, KU Leuven, Ghent, Belgium; ^4^Department of Electrical Engineering and Electronics, University of Liverpool, Liverpool, United Kingdom

**Keywords:** plasma activated liquid, cold atmospheric plasma, influencing factors, planktonic cells, biofilms

## Abstract

Recent research has proven the ability of cold atmospheric plasma (CAP) for assuring food safety. A more flexible and transportable alternative is the use of plasma activated liquids (PAL), which are also known to have antimicrobial properties. However, within the context of food safety, little is known on its potential regarding decontamination. This research therefore focusses on identifying the impact of (i) the microbial species and its cell type (planktonic cells or biofilms), (ii) the CAP settings (i.e., gas composition and generation time) and (iii) PAL related factors (treatment time and PAL age) on the technologies efficacy. Cell densities were monitored using the plate counting technique for which the results were analyzed by means of predictive inactivation models. Moreover, the pH and the concentrations of long-lived species (i.e., hydrogen peroxide, nitrite, and nitrate) were measured to characterize the PAL solutions. The results indicated that although the type of pathogen impacted the efficacy of the treatment, mainly the cell mode had an important effect. The presence of oxygen in the operating gas ensured the generation of PAL solutions with a higher antimicrobial activity. Moreover, to ensure a good microbial inactivation, PAL generation times needed to be sufficiently long. Both CAP related factors resulted in a higher amount of long-lived species, enhancing the inactivation. For 30 min. PAL generation using O_2_, this resulted in log reductions up to 3.9 for biofilms or 5.8 for planktonic cells. However, loss of the PAL activity for stored solutions, together with the frequent appearance of a tailing phase in the inactivation kinetics, hinted at the importance of the short-lived species generated. Different factors, related to (i) the pathogen and its cell mode, (ii) the CAP settings and (iii) PAL related factors, proved to impact the antimicrobial efficacy of the solutions and should be considered with respect to future applications of the PAL technology.

## Introduction

Over the last century, the food industry has invested a significant amount of money and effort in research concerning food safety. With respect to microbiological safety, the occurrence of pathogens in food products or on direct contact surfaces can be a real threat for human health. With respect to treatment of food products, conventional decontamination technologies often cause unwanted side effects on organoleptic, nutritional and functional properties. Especially with an increasing consumption of fresh produce, there is an increasing requirement for milder preservation technologies (Rico et al., 2007). Nowadays, decontamination of fresh produce includes washing combined with chemical biocides (Aharoni et al., 1997; Goodburn and Wallace, 2013). For food contact surfaces, current disinfection processes often involve rinsing with (hot) water and antimicrobial agents, together with a mechanical action (Kim and Wei, 2012). First of all, adverse effects related to the use of chemicals in the above techniques include the formation of toxic and carcinogenic residues (Kumar and Anand, 1998; Simoes et al., 2010; Giaouris et al., 2014). Secondly, most of these techniques rely upon their potential to inactivate planktonic cells and, while many human pathogens were proven to grow predominantly as highly resistant biofilms, they can thus be highly ineffective when applied to inactivate biofilms (Simoes et al., 2010; Hassan et al., 2011; Gómez-López, 2012; Giaouris et al., 2014; Ziuzina et al., 2015). These disadvantages have stimulated research focusing on physical decontamination technologies (Rico et al., 2007; Lopez-Galvez et al., 2013).

One promising physical decontamination technology is the use of cold atmospheric plasma (CAP) (Laroussi et al., 2000). By applying a voltage to a gas stream plasma is generated, resulting in a mixture of charged particles (electrons and ions), reactive species [reactive oxygen and reactive nitrogen species (RNS)], UV photons and electric fields, all characterized by their antimicrobial action (Deng et al., 2006; Misra et al., 2016). While different types of plasma exist based on the conditions under which they are generated, the cold plasmas operating around room temperature at atmospheric pressure CAP are suitable for treatment of food products and food contact surfaces (Misra et al., 2011). CAP has proven to successfully inactivate different bacteria, fungi, viruses and spores for different cell modes of living (i.e., planktonic cells, colonies, and biofilms) (Kelly-Wintenberg et al., 1999; Korachi et al., 2010; Sun et al., 2012; Ziuzina et al., 2015; Smet et al., 2017; Govaert et al., 2018b). Other advantages include (i) short treatment times, (ii) low energy need, and (iii) the fact that chemical species formed during CAP treatment are highly reactive and short-lived, so no residues remain on the surface of the treated product (Moisan et al., 2001). Although its beneficial effect for assuring food safety was proved, the requirement of an on-site production might limit the application of the CAP on an industrial level.

Recent research indicates that plasma activated liquids (PAL) have an antimicrobial action as well. PAL is an indirect mode of application, in which the sample is exposed to CAP pre-treated solutions. The efficacy of the PAL technology is based on the reactive oxygen and RNS generated near the gas-liquid interface, which are transported into the liquid (e.g., water) (Lu et al., 2017). Resulting their dissolution into the liquid, the stable long-lived species in the PAL system (i.e., mainly hydrogen peroxide, nitrite, and nitrate) will be responsible for the microbial inactivation. In addition, the acidification of the liquid can contribute to this inactivation (Jablonowski and von Woedtke, 2015). The PAL technology has the additional advantages of having a high flexibility due to the possibility of production off-site in combination with its transportability and storability (Lu et al., 2017). By means of these advantages, PAL can be considered as a direct sustainable alternative (i) to the washing solutions amended nowadays for treatment of fresh produce, or (ii) to traditional disinfection processes used for food contact surfaces, but without the risk of toxic or carcinogenic by-products formation like associated with the application of chlorine based products (Ma et al., 2015). Of course, a full examination of the complex plasma chemistry and formation of several ROS and RNS is needed to conclude on the final toxicity of the different reaction compounds, and to treat it as generally regarded as safe (GRAS) from a regulatory point of view (Thirumdas et al., 2018).

While the potential of PAL for microbial decontamination is recognized, its application for treatment of food products and food contact surfaces is mostly uninvestigated. Therefore, some basic research challenges and questions remain with respect to inactivation of important food pathogens and the impact of their cell mode. Moreover, with many CAP and PAL related factors determining the efficacy of the treatment, a thorough characterization and validation of PALs on lab scale is required. In this work, the efficacy of PAL for inactivation of *Listeria monocytogenes* and *Salmonella* Typhimurium was assessed while altering (i) the cell type for both bacterial species and (ii) the conditions during PAL generation and treatment. For both bacterial species, two different modes of living were used, i.e., the planktonic form (cells in suspension) and the biofilm cell type (a sessile community of cells embedded in a matrix). For the generation of the PAL, different CAP variables (gas composition and generation time) where altered, while during the consecutive PAL inactivation treatment, the treatment time and the age of the solution (under optimal PAL generation conditions) were considered.

## Materials and Methods

### Experimental Design

The efficacy of PAL for inactivation of both *L. monocytogenes* (Gram-positive) and *S.* Typhimurium (Gram-negative) planktonic cells and biofilms was assessed. For the generation of the PAL, sterile demineralized water was treated with CAP, considering different generation times (i.e., 10, 20, and 30 min) and different CAP gas compositions [i.e., helium with either 0.0 or 1.0 (v/v) % of oxygen]. During PAL treatment, sample treatment times were varied from 0 to 30 min. For the most optimal PAL generation condition, also PAL solutions of a different age (0, 3, 10, and 30 days) were considered. Following PAL treatment, cell densities were determined on both general and selective media. For each experimental condition assessed, two independent biological replicates were used for each (PAL treatment) time point.

For each of the PAL generation conditions, the concentration of three (long-living) PAL species (i.e., hydrogen peroxide, nitrite, and nitrate) was determined immediately after PAL generation in order to link their presence to the observed inactivation efficacy. For the most promising combination of PAL generation conditions (i.e., with the highest inactivation efficacy), the concentration of the PAL species was investigated as well as function of the PAL storage time (i.e., following 0, 3, 10, and 30 days of incubation at 20°C). Finally, the pH of the different PAL solutions was measured as well to possibly link the effect of the pH to the observed inactivation. As for the characterization of the PAL species, the pH was measured for all conditions immediately after the PAL generation, but as function of storage time only for the most promising PAL generation condition. Regarding the PAL characterization, for each of the PAL generation conditions (and each storage time), three independent replicates were performed.

### Microorganisms and Pre-culture Conditions

In this research, biofilm forming strains *L. monocytogenes* LMG 23775 and *S.* Typhimurium LMG 14933 were used. These bacterial species were both acquired from the BCCM/LMG bacteria collection of Ghent University in Belgium and stock-cultures were, respectively, stored at −80°C (U101 Innova, New Brunswick Scientific Co., United States) in brain heart infusion broth (BHI, VWR International, Belgium) and tryptic soy broth (TSB, Becton Dickson, United States), both supplemented with 20 (v/v) % glycerol (VWR International, Belgium).

For every experiment, a purity plate was prepared by spreading a loopful of stock-culture onto an agar plate [lennox luria bertani agar (Becton Dickinson, United States) supplemented with 5 g/L NaCl (Sigma-Aldrich, United States)]. Agar plates were incubated (Binder BD115, VWR International, Belgium) for 24 h at the most optimal growth temperature for these microorganisms, i.e., 30 and 37°C for *L. monocytogenes* and *S.* Typhimurium, respectively (Belgian Co-ordinated Collections of Micro-organisms [BCCM], 2017). Pre-cultures were prepared by transferring one colony from the incubated purity plate into an Erlenmeyer containing 20 mL of fresh growth medium [lennox luria bertani broth (Becton Dickinson, United States) supplemented with 5 g/L NaCl]. Pre-cultures were again incubated for 24 h at 30 (*L. monocytogenes*) or 37°C (*S.* Typhimurium). After incubation, stationary phase pre-cultures were obtained with a cell density of approximately 10^9^ CFU/mL.

### Biofilm Development

For the development of the biofilm cell type, the protocol and optimal incubation conditions of Govaert et al. (2018a) were used to obtain strongly adherent and mature biofilms. Biofilms were grown on polystyrene Petri dishes, representing hydrophobic surfaces relevant for the food industry (cutting surfaces made of polymeric material, packaging materials, conveyor belts, tanks, pipework, etc.). In summary, the stationary phase pre-cultures were 100-fold diluted in BHI and in 20-fold diluted TSB for *L. monocytogenes* and *S.* Typhimurium, respectively. This inoculum was used to inoculate small polystyrene Petri dishes (50 mm diameter, 9 mm height, Simport, Canada) with 1.2 mL of the cell suspension, after which the Petri dishes were closed and gently shaken to make sure the inoculum covered the entire surface. Finally, Petri dishes were incubated for 24 h at 30 (*L. monocytogenes*) or 25°C (*S.* Typhimurium), resulting in mature biofilms with a cell density of approximately 10^6^–10^7^ CFU/cm^2^.

### CAP Equipment and PAL Generation

**FIGURE 1:**
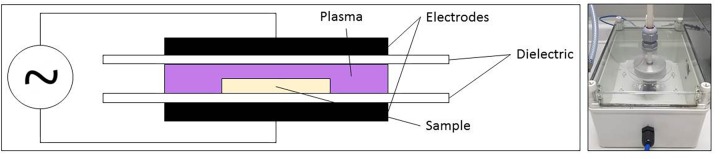
CAP set-up for plasma activated liquid generation.

For the generation of the PAL, sterile demineralized water (3 mL) was treated using CAP. The CAP set-up applied for this research, a DBD electrode, was represented in Figure 1. With this set-up, the discharge is generated between two electrodes (diameter = 5.5 cm and gap = 0.8 cm), covered by a dielectric layer (diameter = 7.5 cm). Around both electrodes, an enclosure was provided to increase the residence time of the plasma species and to obtain a more controlled environment. However, the enclosure was not airtight, which resulted in the presence of low amounts of oxygen and nitrogen from the environment. For all experiments, the CAP was generated in a gas mixture of helium (purity 99.996%, at a flow rate of 4 L/min) and oxygen (purity ≥ 99.995%). Two different oxygen levels were tested, i.e., 0.0 and 1.0 (v/v) %, resulting in oxygen flow rates of 0 and 40 mL/min, respectively. The helium and oxygen flow rates were mixed before entering the plasma enclosure. The plasma power supply transforms a low voltage DC input (21.88 V) into a high voltage AC signal (approximately 8 kV), at a frequency of 15 kHz. The input voltage was selected based on the studies of Smet et al. (2017) and Govaert et al. (2018b), since the highest CAP inactivation efficacy (for planktonic cells and biofilms) was obtained while using this voltage.

After placing the sample (water containing Petri dish) between the electrodes, the reactor chamber was flushed (4 min) to ensure a homogeneous gas composition in the enclosure, and the samples were CAP treated for either 10, 20, or 30 min. The generated PAL solutions were characterized and used for treatment of the *S.* Typhimurium and *L. monocytogenes* planktonic cells and biofilms.

### PAL Characterization

To characterize the composition of the generated PAL solutions, the concentration of three important (long-living) PAL species (i.e., hydrogen peroxide, nitrite, and nitrate) was determined in order to possibly link their presence to the observed inactivation efficacy. For each of the PAL generation conditions [i.e., 10/20/30 min of CAP treatment using a gas flow containing either 0 or 1 (v/v) % oxygen], the concentration of these PAL species was determined immediately after PAL generation. For the most promising combination of PAL generation conditions (i.e., the one with the highest inactivation efficacy), the PAL composition was also assessed as function of the storage time (i.e., following 0, 3, 10, and 30 days of storage at 20°C).

To determine the hydrogen peroxide (H_2_O_2_) concentration, a colorimetric method involving the use of Titanium Oxysulfate (TiOSO_4_) was used. Here, 100 μL of the PAL solution was added to a well of a 96-well microtiter plate containing 10 μL TiOSO_4_. This mixture was incubated for 10 min at room temperature and in the dark. Following this incubation period, a microplate reader (VersaMax tunable microplate reader, Molecular devices, United Kingdom) was used to measure the absorbance at 405 nm. In addition, a standard curve of known H_2_O_2_ concentrations was developed and used to convert absorbance values into H_2_O_2_ concentrations. If necessary, PAL samples were diluted to fit the concentration range of the calibration curve (data not shown). To prepare the H_2_O_2_ calibration curve, a 30 % hydrogen peroxide standard solution (Sigma-Aldrich, United States) was diluted to obtain standard solutions with the following concentrations: 0, 2 × 10^−4^, 3 × 10^−4^, 5 × 10^−4^, 1 × 10^−3^, 2 × 10^−3^, 3 × 10^−3^, and 5 × 10^−3^ % (1% = 0.4263 M). To develop the calibration curve, average values of three replicates were used to determine the equation of the absorbance as function of the H_2_O_2_ concentration.

To measure the nitrite/nitrate (NO_2_^−^/NO_3_^−^) concentration of the different PAL solutions, another colorimetric method was used. Here, the nitrite/nitrate Assay kit (Sigma-Aldrich, United States) was used as recommended in the corresponding manual. To determine the amount of nitrite, 100 μL of the PAL solution was added to a well of a 96-well microtiter plate containing 50 μL of Griess reagent A. These solutions were mixed (MS3D, IKA, Germany) and incubated at 25°C for 5 min. Following these 5 min of incubation, 50 μL of Griess reagent B was added, the solutions were mixed, and incubated at 25°C for 10 min. Finally, the absorbance was measured with the microplate reader at 540 nm. To determine the amount of nitrate, the amount of nitrate present in 80 μL of PAL solution was first reduced to nitrite by adding 10 μL of nitrate reductase and 10 μL of enzyme co-factor solution. Following 2 h of incubation at 25°C, 50 μL of Griess reagent A was added. This mixture was mixed and incubated at 25°C for 5 min. Next, 50 μL Griess reagent B was added, the solutions were again mixed, and incubated at 25°C for 10 min. Finally, the absorbance was measured at 540 nm to determine the total concentration of nitrite+nitrate. Consequently, the amount of nitrate present in the PAL solution was calculated by subtracting the nitrite amount from the total nitrite+nitrate concentration. To convert measured absorbance into concentrations, for both the nitrite and the total amount of nitrite+nitrate, a calibration curve was developed (data not shown, based on average values of three replicates). These curves were made as described in the protocol of the nitrite/nitrate assay kit.

To examine the effect of the PAL generation conditions on the pH of the PAL solutions (and their inactivation efficacy), the pH was measured (SevenCompact, Mettler Toledo, Belgium) for all PAL generation conditions and storage times.

### PAL Inactivation and Microbial Analysis

Depending on the type of cells, a different PAL inactivation treatment protocol was implemented. Moreover, PAL solutions of a different age, i.e., stored in falcon tubes for 0, 3, 10, or 30 days at 20°C, were used.

For the planktonic cells, the 3 mL of PAL within the falcon tube was inoculated with 30 μL of the pre-culture (with a cell density of approximately 10^9^ CFU/mL) to finally obtain a starting cell density of approximately 10^7^ CFU/mL. Following 5, 10, 25, or 30 min of PAL treatment, 1 mL of the cell suspension was transferred from the falcon tube to an empty sterile Eppendorf. Immediately, serial decimal dilutions were prepared and plated on both general and selective medium. For the untreated planktonic cells (0 min PAL treatment time), 30 μL of the pre-culture was added to 3 mL of sterile demineralized water. This obtained suspension was diluted and plated on the same media as for the inoculated PAL solutions.

For cells grown as biofilms, the 24 h old biofilms were first rinsed 3 times with 1.2 mL of sterile phosphate buffered saline (PBS) solution in order to remove the remaining planktonic cells. After drying, 1.2 mL of the PAL from the falcon tubes was added to the Petri dishes. After 5, 10, 25, or 30 min of PAL treatment, the PAL was removed, and the biofilms were rinsed 3 times with sterile PBS solution in order to remove all remaining active species. After this, biofilms were again allowed to dry and the cell scraping method as described in Govaert et al. (2018a) was used to remove the biofilms from the surface. Finally, serial decimal dilutions were made from the obtained cell suspensions and plated on both general and selective medium. For the untreated biofilms (0 min PAL treatment time), the cell scraping method (followed by making serial decimal dilutions and plating) was used immediately after the first rinsing and drying step.

For both microorganisms and both types of cells, serial decimal dilutions were prepared in 0.85 (m/v) % NaCl solution. For plate counts, BHI agar (BHIA, BHI supplemented with 14 g/l agar, VWR Chemicals, Belgium) and PALCAM agar (VWR Chemicals, Belgium) were used for *L. monocytogenes*, while tryptic soy agar (TSA, TSB supplemented with 14 g/l agar) and xylose lysine deoxycholate agar (XLDA, Merck & Co., United States) were used for *S.* Typhimurium. Three drops (20 μL/drop) of each serial dilution were plated on both media for each microorganism (Miles et al., 1938). Before counting the colonies, BHI and PALCAM agar plates were incubated for (at least) 24 h at 30°C and TSA and XLD agar plates were incubated for 24 h at 37°C. The detection limit of this assay was around 1.7 log(CFU/cm^2^) for biofilms and around 2.6 log(CFU/mL) for planktonic cells.

### Modeling, Parameter Estimation, and Estimation of Sublethal Injury

The model of Geeraerd et al. (2000) (without a shoulder phase) was used to fit the experimental data. This model describes a microbial inactivation curve consisting of a log-linear inactivation phase and a tail (Equation 1).

$$N(t)=(N_{0}-N_{\mathrm{res}})\times e^{-k_{\max}\times t}+N_{\mathrm{res}}$$

Where *N(t)* [CFU/mL (planktonic cells) or CFU/cm^2^ (biofilms)] is the cell density at time t [min], *N*_0_ [CFU/mL (planktonic cells) or CFU/cm^2^ (biofilms)] the initial cell density, *N*_res_ [CFU/mL (planktonic cells) or CFU/cm^2^ (biofilms)] is a more resistant subpopulation, and *k*_max_ [1/min] the maximum specific inactivation rate. In the rare case no tail was present (*N*_res_ = 0), the model was reduced to a log-linear fit. Based on the difference between log10 *N*_0_ and log_10_ *N*_res_, the final log-reduction was calculated for each combination of oxygen level, CAP treatment time, and microbial cell type.

The parameters of Geeraerd et al. (2000) model were estimated via the minimization of the sum of squared errors (SSE), using the lsqnonlin routine of the Optimization Toolbox of Matlab version R2015b (The Mathworks, Inc.). At the same time, the parameter estimations were determined based on the Jacobian matrix. The Root Mean Squared Error (RMSE) served as an absolute measure of the goodness of the model to fit the actual obtained data.

Finally, to calculate the percentage of sublethal injury (% SI), theoretical concentrations obtained from the model of Geeraerd et al. (2000) were used for both the general (BHI/TSA) and selective (PALCAM/XLD) counts. The equation of Busch and Donnelly (1992) (Equation 2) was used to determine the percentage of injured cells at each PAL treatment time. As a result, the percentage of sublethal injury was plotted as function of the PAL treatment time.

$$\%\mathrm{SI}\frac{\mathrm{CFU general medium}-\mathrm{CFU selective medium}}{\mathrm{CFU general medium}}\cdot 100$$

### Statistical Analysis

The analysis of variance (ANOVA) test was performed to determine whether there are any significant differences among (i) means of the PAL characteristics (Table 1) or (ii) the estimated Geeraerd et al. (2000) model parameters (Tables 2–4), at a 95.0% confidence level (α = 0.05). Fisher’s Least Significant Difference (LSD) test was used to distinguish which means were significantly different from which others. Standardized skewness and standardized kurtosis were used to assess if data sets came from normal distributions. These analyses were performed using the Statgraphics Centurion XVI.I Package (Statistical Graphics, WA, United States). Test statistics were regarded as significant when *P* was ≤ 0.05.

**Table 1:** Characterization of plasma activated liquids.

Gas composition	Generation time	PAL age	PAL characterization parameter			
			^1^pH (−)^2^_3_	^1^H_2_O_2_ (μM)^2^_3_	^1^NO_2_^−^ (μM)^2^_3_	^1^NO_3_^−^ (μM)^2^_3_
0% (v/v) O_2_	10 min	0 days	^2^3.61 ± 0.06^2^	^1^588.9 ± 106.8^1^	^1^25.5 ± 17.3^2^	^1^49.9 ± 6.1^1^
	20 min	0 days	^1^3.19 ± 0.14^1^	^1^1215.8 ± 73.7^2^	^1^1.5 ± 1.9^1^	^1^66.8 ± 7.9^2^
	30 min	0 days	^1^3.10 ± 0.04^1^	^1^1875.7 ± 239.2^3^	^1^2.0 ± 3.3^1^	^1^61.3 ± 10.0^1,2^
1% (v/v) O_2_	10 min	0 days	^1^3.28 ± 0.13^2^	^1^1932.7 ± 1865.0^1^	^1^4.7 ± 7.5^1^	^1^59.6 ± 6.9^1^
	20 min	0 days	^1^3.14 ± 0.16^1,2^	^1^1376.1 ± 393.4^1^	^1^1.2 ± 2.5^1^	^1^63.3 ± 13.3^1^
	30 min	0 days	^1^2.94 ± 0.13^1^_A_	^1^1963.6 ± 671.0^1^_A_	^1^1.1 ± 2.4^1^_A_	^1^57.3 ± 15.9^1^_A_
		3 days	4.25 ± 1.00_B_	2216.6 ± 900.8_A_	0.0 ± 0.0_A_	67.7 ± 25.1_A_
		10 days	2.93 ± 0.17_A_	2289.7 ± 992.6_A_	0.0 ± 0.0_A_	51.8 ± 22.9_A_
		30 days	2.92 ± 0.14_A_	2733.9 ± 798.7_A_	0.0 ± 0.0_A_	84.6 ± 12.4_A_

*^1^Effect gas composition: for each generation time and PAL age, PAL characterization parameters bearing different superscripts (no numbers in common) are significantly different (P ≤ 0.05). ^2^Effect generation time: for each gas composition and PAL age, PAL characterization parameters bearing different superscripts (no numbers in common) are significantly different (P ≤ 0.05). ^3^Effect PAL age: for each gas composition and generation time, PAL characterization parameters bearing different subscripts (no uppercase letters in common) are significantly different (P ≤ 0.05).*

**Table 2:** Inactivation parameters of Geeraerd et al. (2000) model for *L. monocytogenes* planktonic cells and biofilms after plasma activated liquid treatment.

Cell type	Gas composition	Generation time	Population	Inactivation parameters			RMSE	^1^log reduction^2^_3_
				^1^log N_0_ (log [CFU/mL)]^2^_3_/ ^1^log N_0_ [log(CFU/cm^2^)]^2^_3_	^1^k_max_ (1/min)^2^_3_	^1^log N_res_ [log(CFU/mL)]^2^_3_ / ^1^logN_res_ [log(CFU/cm^2^)]^2^_3_		
Planktonic	0% (v/v) O_2_	10 min	Total	6.8 ± 0.0^2^_A_	0.003 ± 0.004^1^_A_	–	0.0583	≈ ^1^0.0 ± 0.0^1^_A_
cells			Uninjured	6.8 ± 0.0^2^_A_	^1^0.001 ± 0.003^1^_A_	–	0.0716	≈ ^1^0.1 ± 0.0^1^_A_
		20 min	Total	6.8 ± 0.0^1^_A_	0.004 ± 0.004^1^_A_	–	0.0955	≈ ^1^0.0 ± 0.0^1^_A_
			Uninjured	6.7 ± 0.1^1^_A_	^1^0.000 ± 0.005_A_	–	0.2739	≈ ^1^0.5 ± 0.1^1^_B_
		30 min	Total	6.9 ± 0.0^1^_B_	^1^0.042 ± 0.014^1^_B_	–	0.0665	≈ ^1^0.1 ± 0.0^1^_B_
			Uninjured	6.8 ± 0.1^1^_A_	^1^0.009 ± 0.003_B_	–	0.2056	≈ ^1^0.4 ± 0.1^1^_B_
	1% (v/v) O_2_	10 min	Total	6.7 ± 0.0^1^_A_	^1^0.030 ± 0.010^2^_A_	–	0.0847	≈ ^1^0.0 ± 0.0^1^_A_
			Uninjured	6.6 ± 0.1^1^_A_	^1^0.021 ± 0.016^1^	–	0.3136	≈ ^1^0.3 ± 0.1^2^_A_
		20 min	Total	6.8 ± 0.5^1^_A_	^2^1.797 ± 0.332^2^_B_	2.0 ± 0.3	0.9620	^2^4.8 ± 0.6^2^_B_
			Uninjured	6.9 ± 0.5^1^_A_	20.000 inf	1.2 ± 0.2	0.9430	^2^5.7 ± 0.5^2^_B_
		30 min	Total	6.8 ± 0.7^1^_A_	2.572 ± 0.810^2^_B_	1.3 ± 0.4	1.3619	^2^5.5 ± 0.8^2^_B_
			Uninjured	6.5 ± 0.5^1^_A_	19.272 inf	1.2 ± 0.3	1.0609	^2^5.3 ± 0.6^2^_B_
Biofilms	0% (v/v) O_2_	10 min	Total	7.2 ± 0.1^1^_A_	5.421 inf	5.4 ± 0.1^1^_A_	0.3643	^2^1.8 ± 0.1^2^_AB_
			Uninjured	7.1 ± 0.1^1^_A,B_	^2^4.852 ± 0.000^2^_C_	5.4 ± 0.1^1^_A_	0.3990	^2^1.7 ± 0.1^1^_A_
		20 min	Total	7.3 ± 0.1^1^_A_	5.563 inf	5.3 ± 0.1^1^_A_	0.3267	^2^2.0 ± 0.1^1^_B_
			Uninjured	7.3 ± 0.1^1^_B_	^2^1.120 ± 0.279^2^_B_	5.2 ± 0.1^1^_A_	0.3411	^2^2.1 ± 0.1^1^_B_
		30 min	Total	7.1 ± 0.2^1^_A_	^1^0.602 ± 0.239	5.5 ± 0.2^2^_A_	0.6065	^2^1.6 ± 0.3^1^_A_
			Uninjured	7.0 ± 0.2^1^_A_	^2^0.457 ± 0.155_A_	5.2 ± 0.2^2^_A_	0.6492	^2^1.8 ± 0.3^1^_AB_
	1% (v/v) O_2_	10 min	Total	7.2 ± 0.1^1^_A_	^2^0.411 ± 0.087	5.7 ± 0.1^2^_C_	0.3276	^2^1.5 ± 0.1^1^_A_
			Uninjured	7.1 ± 0.1^1^_A_	^2^0.392 ± 0.099^1^	5.6 ± 0.1^1^_C_	0.3823	^2^1.5 ± 0.1^1^_A_
		20 min	Total	7.4 ± 0.1^1^_B_	^1^0.520 ± 0.118	5.1 ± 0.2^1^_B_	0.5916	^1^2.3 ± 0.2^1^_B_
			Uninjured	7.3 ± 0.2^1^_A_	0.571 ± 0.131^1^	4.9 ± 0.2^1^_B_	0.6566	^1^2.4 ± 0.3^1^_B_
		30 min	Total	7.3 ± 0.1^1^_A,B_	6.017 inf	4.3 ± 0.1^1^_A_	0.4363	^1^3.0 ± 0.1^2^_C_
			Uninjured	7.2 ± 0.1^1^_A_	6.022 inf	4.0 ± 0.1^1^_A_	0.5238	^1^3.2 ± 0.1^2^_C_

*Plasma activated liquids were created using different cold atmospheric plasma gas compositions and generation times. Inactivation parameters are determined for both the total and the uninjured population. ^1^Effect cell mode: for each gas composition, generation time and population type, parameters of the Geeraerd et al. (2000) model bearing different superscripts (no numbers in common) are significantly different (P ≤ 0.05). ^2^Effect gas composition: for each cell mode, generation time and population type, parameters of the Geeraerd et al. (2000) model bearing different superscripts (no numbers in common) are significantly different (P ≤ 0.05). ^3^Effect generation time: for each cell mode, gas composition and population type, parameters of the Geeraerd et al. (2000) model bearing different subscripts (no uppercase letters in common) are significantly different (P ≤ 0.05).*

## Results

### PAL Characteristics

Before treatment, the pH of sterile deionized water was in the range of 6.67 to 6.80. In general, when generating PAL in water, the pH decreased from neutral to acidic (Table 1). This drop was dependent on the different CAP factors selected during the generation of the PAL solution. The CAP operating gas was important, as indicated by (significant) lower pH values obtained in the presence of oxygen. For the PAL generation time, it can be observed that the pH further significantly decreased with an increasing generation time. During storage of the PAL solutions, the pH of the PAL solutions proved constant.

Regarding H_2_O_2_, its concentration increased with generation time while no significant differences were observed related to the CAP operating gas. Moreover, following storage of the solutions, relatively constant concentrations of hydrogen peroxide were observed.

Although Table 1 indicated that nitrite was barely detected in the PAL samples, nitrate was observed at higher concentrations. However, the different factors (i.e., gas composition, generation time, and storage) appear to have a limited impact on the NO_3_^−^ concentration.

### Influence of Microbial Species and Type of Cells

**FIGURE 2:**
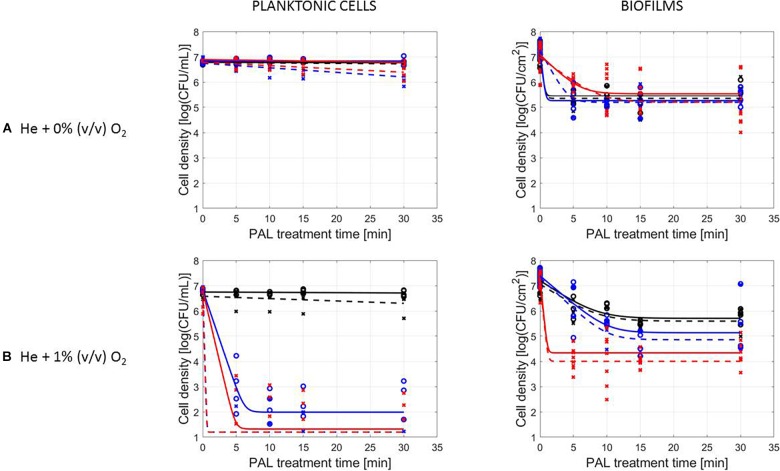
Survival curves of *L. monocytogenes* planktonic cells and biofilms after plasma activated liquid treatment. Plasma activated liquids were created using two different cold atmospheric plasma gas compositions **(A)** He + 0% (v/v) O_2_ or **(B)** He + 1% (v/v) O_2_, and three different generation times (10, 20, and 30 min). Experimental data (symbols) and global fit (line) of Geeraerd et al. (2000) model: total viable population (o, solid line) and uninjured viable population (x, dashed line).

**FIGURE 3:**
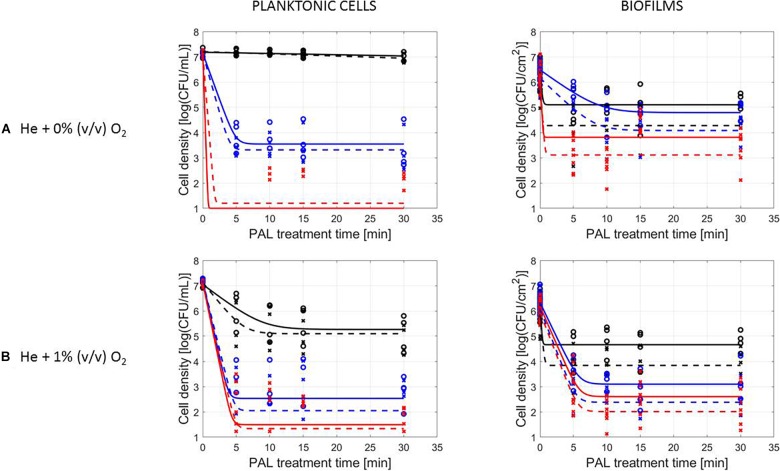
Survival curves of *S.* Typhimurium planktonic cells and biofilms after plasma activated liquid treatment. Plasma activated liquids were created using two different cold atmospheric plasma gas compositions **(A)** He + 0% (v/v) O_2_ or **(B)** He + 1% (v/v) O_2_, and three different generation times (10, 20, and 30 min). Experimental data (symbols) and global fit (line) of Geeraerd et al. (2000) model: total viable population (o, solid line) and uninjured viable population (x, dashed line).

The PAL efficacy highly depended on the microbial species. As indicated in Figure 2, 3 (PAL age = 0 days), *L. monocytogenes* exhibited a higher resistance to PAL compared to *S.* Typhimurium. This was confirmed by the calculated log-reductions in Tables 2, 3. However, the PAL inactivation efficacy was also highly influenced by the different factors assessed in this manuscript, as indicated by both Figure 2, 3 and Tables 2, 3.

**Table 3:** Inactivation parameters of Geeraerd et al. (2000) model for *S.* Typhimurium planktonic cells and biofilms after plasma activated liquid treatment.

Cell type	Gas composition	Generation time	Population	Inactivation parameters			RMSE	^1^log reduction^2^_3_
				^1^log N_0_ (log [CFU/mL)]^2^_3_ / ^1^log N_0_ [log(CFU/cm^2^)]^2^_3_	^1^k_max_ (1/min)^2^_3_	^1^log N_res_ [log(CFU/mL)]^2^_3_ / ^1^logN_res_ [log(CFU/cm^2^)]^2^_3_		
Planktonic cells	0% (v/v) O_2_	10 min	Total	7.2 ± 0.0^1^_A_	0.011 ± 0.006^1^	–	0.1255	≈ ^1^0.2 ± 0.0^1^_A_
			Uninjured	7.2 ± 0.0^1^_A_	0.020 ± 0.006^1^	–	0.1276	≈ ^1^0.3 ± 0.0^1^_A_
		20 min	Total	7.2 ± 0.3^1^_A_	^2^1.686 ± 0.323^1^	3.5 ± 0.2^2^	0.5676	^2^3.7 ± 0.4^1^_B_
			Uninjured	7.1 ± 0.3^1^_A_	^1^2.159 ± 1.145^1^	3.3 ± 0.2^2^	0.5250	^2^3.8 ± 0.4^1^_B_
		30 min	Total	7.2 ± 0.6^1^_A_	17.659 inf	1.0 ± 0.3^1^	1.1470	^2^6.2 ± 0.7^1^_C_
			Uninjured	7.1 ± 0.5^1^_A_	7.592 inf	1.2 ± 0.3^1^	1.0927	^2^5.9 ± 0.6^1^_C_
	1% (v/v) O_2_	10 min	Total	7.0 ± 0.3^1^_A_	0.463 ± 0.186^2^_A_	5.3 ± 0.2_C_	0.6393	^1^1.7 ± 0.4^2^_A_
			Uninjured	7.1 ± 0.3^1^_A_	0.762 ± 0.279^2^_A_	5.1 ± 0.2_C_	0.6832	^1^2.0 ± 0.4^2^_A_
		20 min	Total	7.2 ± 0.7^1^_A_	^1^2.779 ± 8.634^1^_A_	2.5 ± 0.4^1^_B_	1.3344	^2^4.7 ± 0.8^1^_B_
			Uninjured	7.2 ± 0.7^1^_A_	^1^2.495 ± 1.028^1^_B_	2.0 ± 0.4^1^_B_	1.3610	^2^5.2 ± 0.8^1^_B_
		30 min	Total	7.1 ± 0.5^1^_A_	^1^2.962 ± 1.995_A_	1.5 ± 0.3^1^_A_	1.1517	^2^5.6 ± 0.6^1^_B_
			Uninjured	7.1 ± 0.5^1^_A_	^2^2.667 ± 0.615_B_	1.3 ± 0.3^1^_A_	1.0945	^2^5.8 ± 0.6^1^_B_
Biofilms	0% (v/v) O_2_	10 min	Total	6.4 ± 0.1^1^_AB_	7.650 inf	5.1 ± 0.1^2^_C_	0.4905	^2^1.3 ± 0.1^1^_A_
			Uninjured	5.9 ± 0.2^1^_A_	10.683 inf	4.3 ± 0.2^2^_B_	0.6235	^2^1.6 ± 0.3^1^_A_
		20 min	Total	6.5 ± 0.1^1^_B_	^1^0.441 ± 0.094^1^	4.8 ± 0.1^2^_B_	0.3836	^1^1.7 ± 0.1^1^_B_
			Uninjured	6.2 ± 0.1^1^_B_	^1^0.585 ± 0.145^1^	4.1 ± 0.2^2^_B_	0.5457	^1^2.1 ± 0.2^1^_B_
		30 min	Total	6.3 ± 0.1^1^_A_	12.753 inf	3.8 ± 0.1^2^_A_	0.5534	^1^2.5 ± 0.1^1^_C_
			Uninjured	6.0 ± 0.1^1^_AB_	8.823 inf	3.1 ± 0.1^2^_A_	0.5698	^1^2.9 ± 0.1^1^_C_
	1% (v/v) O_2_	10 min	Total	6.3 ± 0.1^1^_A_	11.335 inf	4.7 ± 0.1^1^_C_	0.5150	^1^1.6 ± 0.1^2^_A_
			Uninjured	5.9 ± 0.2^1^_A_	6.925 inf	3.8 ± 0.2^1^_C_	0.6264	^1^2.1 ± 0.3^1^_A_
		20 min	Total	6.3 ± 0.1^1^_A_	^1^1.269 ± 0.158^2^	3.1 ± 0.1^1^_B_	0.4751	^1^3.2 ± 0.1^2^_B_
			Uninjured	6.1 ± 0.1^1^_A_	^1^1.560 ± 0.202^2^	2.4 ± 0.2^1^_B_	0.5521	^1^3.7 ± 0.2^2^_B_
		30 min	Total	6.2 ± 0.1^1^_A_	^1^1.403 ± 0.160	2.6 ± 0.1^1^_A_	0.5000	^1^3.6 ± 0.1^2^_C_
			Uninjured	5.9 ± 0.1^1^_A_	^1^1.579 ± 0.174	2.0 ± 0.2^1^_A_	0.5330	^1^3.9 ± 0.2^2^_B_

*Plasma activated liquids were created using different cold atmospheric plasma gas compositions and generation times. Inactivation parameters are determined for both the total and the uninjured population. ^1^Effect cell mode: for each gas composition, generation time and population type, parameters of the Geeraerd et al. (2000) model bearing different superscripts (no numbers in common) are significantly different (P ≤ 0.05). ^2^Effect gas composition: for each cell mode, generation time and population type, parameters of the Geeraerd et al. (2000) model bearing different superscripts (no number in common) are significantly different (P ≤ 0.05). ^3^Effect generation time: for each cell mode, gas composition and population type, parameters of the Geeraerd et al. (2000) model bearing different subscripts (no uppercase letters in common) are significantly different (P ≤ 0.05).*

A first important influencing factor related to the type of cells. When comparing PAL treatment of planktonic cells and biofilms, different inactivation kinetics emerged: while in most cases for both modes of living a log-linear inactivation phase was followed by a tail, some experimental conditions for planktonic cells indicated a limited inactivation sometimes expressed by only a log-linear phase (Figure 2, 3, PAL age = 0 days).

The differences between the two types of cells were most expressed for *L. monocytogenes*. Here, at 0% (v/v) O_2_, inactivation of planktonic cells always followed a log-linear inactivation as a function of time, and PAL treatment of biofilms was expressed by a short log-linear inactivation followed by a long tailing phase. Focusing on the inactivation parameters, this results in the knowledge that *k*_max, biofilms_ > *k_max, planktonic cells_*, while the overall *log-reduction* of the biofilms was significantly higher than the reduction obtained for planktonic cells. However, this trend completely changed for conditions where PAL was generated using 1% (v/v) O_2_. Then, except for PAL generation times of 10 min, the log-linear phase for planktonic cells was followed by a tail, and both inactivation rates and *log-reductions* were now in favor of the planktonic cells PAL treatment.

Regarding PAL treatment of *S.* Typhimurium, differences between planktonic cells and biofilms at both gas compositions were less evident, as most of the time a rapid inactivation was followed by a long tail. However, for longer generation times, biofilms exhibited a higher resistance toward PAL treatment, while log reductions for planktonic cells were high. Regarding final *log-reductions*, the wide range observed for planktonic cells highlights the importance of the gas composition and generation times.

As an exception, for (i) 10 min PAL generation times and (ii) 0% (v/v) O_2_, higher inactivation efficiencies were observed for the *S.* Typhimurium and *L. monocytogenes* biofilms compared to planktonic cells.

### Influence of CAP Factors Used for PAL Generation

The results in Figure 2, 3 (PAL age = 0 days), indicated an increase of the PAL efficacy when adding oxygen in the CAP operating gas, both for treatment of *S.* Typhimurium and *L. monocytogenes*, as for treatment of planktonic cells and biofilms.

More specifically, by means of the addition of oxygen during PAL generation, the *L. monocytogenes* inactivation parameters (Table 2) indicated an increase in *k*_max_ and *log-reduction* for planktonic cells. For biofilms, the inactivation parameters (including *N*_res_) were less affected by the gas composition.

Regarding the impact of the gas composition when treating *S.* Typhimurium with PAL, no clear trend was deducted for most inactivation parameters. However, the importance of 1% (v/v) oxygen was apparent by significantly lower *N*_res_ values (and higher *log-reductions*) for treatment of biofilms.

By increasing the PAL generation time, also the antimicrobial activity increased (Figure 2, 3). Regardless the microorganism, the cell type or gas composition during generation, the highest PAL efficacy was obtained for generation times of 30 min. The influence of generation time on the inactivation parameters could again be deducted in Tables 2, 3.

More specifically, for *L. monocytogenes* (both cell modes and gas compositions), both *k*_max_ and the *log-reductions* significantly increased with an extended generation time. *N*_res_ for biofilms treated with 1% (v/v) O_2_ significantly reduced due to a longer generation.

Although for *S.* Typhimurium, the increase in *k*_max_ at longer generation times was only significant for planktonic cells, *log-reductions* also always significantly increased while *N*_res_ decreased.

### Influence of PAL Factors

Figure 2, 3, indicating the cell reduction as a function of PAL treatment time, were previously discussed in detail. Regardless of the other influencing factors, if inactivation was observed, a rapid inactivation phase was shown to be followed by a long tail. This illustrated that although the increase of PAL treatment time would result in a higher efficacy, this increase could only be expected up to a certain time. For both microbial species, this shift toward the tail was highly influenced by the other factors discussed previously.

**FIGURE 4:**
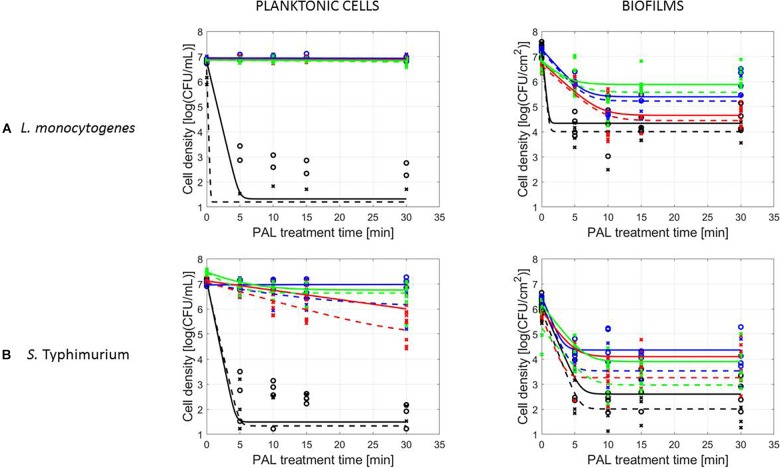
Survival curves of **(A)** *L. monocytogenes* or **(B)** *S.* Typhimurium planktonic cells and biofilms after treatment with plasma activated liquids of different ages (0, 3, 10, and 30 days). Experimental data (symbols) and global fit (line) of Geeraerd et al. (2000) model: total viable population (o, solid line) and uninjured viable population (x, dashed line).

A final influencing factor that was assessed within this research was related to the PAL age, as the solutions were stored up to 1 month to check the influence of age on their efficacy. Based on the previous results (see section “Influence of CAP Factors Used for PAL Generation”), the optimal CAP settings were selected to generate the PAL, i.e., generation for 30 min using helium with 1% (v/v) O_2_. For the 4 different storage times (0, 3, 10, and 30 days), the PAL efficacy on the cell density as a function of PAL treatment time (again up to 30 min) was summarized in Figure 4, while all inactivation parameters were provided in Table 4. Although both *L. monocytogenes* and *S.* Typhimurium followed similar trends, the cell mode proved to impact the efficacy of the treatment, i.e., for the stored PAL solutions, a better result was obtained for treatment of biofilms.

**Table 4:** Inactivation parameters of Geeraerd et al. (2000) model for *L. monocytogenes* and *S.* Typhimurium planktonic cells and biofilms after treatment with plasma activated liquids of different ages.

Microorganism	Cell type	PAL age	Population	Inactivation parameters			RMSE	^1^log reduction^2^
				^1^log N_0_ (log [CFU/mL)]^2^ / ^1^log N_0_ [log(CFU/cm^2^)]^2^	^1^k_max_ (1/min)^2^	^1^log N_res_ [log(CFU/mL)]^2^ / ^1^logN_res_ [log(CFU/cm^2^)]^2^		
*Listeria monocytogenes*	Planktonic cells	0 days	Total	6.8 ± 0.7^1^	2.572 ± 0.810^2^	1.3 ± 0.4	1.3619	^2^5.5 ± 0.8^2^
			Uninjured	6.5 ± 0.5^1^	19.272 inf	1.2 ± 0.3	1.0609	^2^5.3 ± 0.6^2^
		3 days	Total	6.9 ± 0.0^1^	^1^0.001 ± 0.005^1^	–	0.0965	≈ ^1^0.0 ± 0.0^1^
			Uninjured	6.9 ± 0.0^1^	^1^0.001 ± 0.005	–	0.1028	≈ ^1^0.0 ± 0.0^1^
		10 days	Total	6.9 ± 0.0^1^	^1^0.001 ± 0.004^1^	–	0.0742	≈ ^1^0.0 ± 0.0^1^
			Uninjured	6.9 ± 0.0^1^	^1^0.001 ± 0.003	–	0.0656	≈ ^1^0.0 ± 0.0^1^
		30 days	Total	6.9 ± 0.0^1^	^1^0.002 ± 0.004^1^	–	0.0791	≈ ^1^0.1 ± 0.0^1^
			Uninjured	6.9 ± 0.0^1^	^1^0.006 ± 0.004	–	0.0789	≈ ^1^0.1 ± 0.0^1^
	Biofilms	0 days	Total	7.3 ± 0.1^2^	6.017 inf	4.3 ± 0.1^1^	0.4363	^1^3.0 ± 0.1^3^
			Uninjured	7.2 ± 0.1^2^	6.022 inf	4.0 ± 0.1^1^	0.5238	^1^3.2 ± 0.1^3^
		3 days	Total	7.3 ± 0.3^2^	^2^0.703 ± 0.230	5.4 ± 0.2^3^	0.5604	^2^1.9 ± 0.4^2^
			Uninjured	7.2 ± 0.3^2^	^2^0.704 ± 0.236	5.2 ± 0.2^3^	0.6054	^2^2.0 ± 0.4^2^
		10 days	Total	6.8 ± 0.3^1^	^2^0.565 ± 0.187	4.7 ± 0.2^2^	0.6262	^2^2.1 ± 0.4^2^
			Uninjured	6.6 ± 0.3^1^	^2^0.548 ± 0.173	4.4 ± 0.2^2^	0.6310	^2^2.2 ± 0.4^2^
		30 days	Total	6.9 ± 0.3^1,2^	^1^0.588 ± 0.579	5.9 ± 0.2^4^	0.6504	^2^1.0 ± 0.4^1^
			Uninjured	6.8 ± 0.3^1,2^	^2^0.511 ± 0.292	5.6 ± 0.2^4^	0.5645	^2^1.2 ± 0.4^1^
*Salmonella* Typhimurium	Planktonic cells	0 days	Total	7.1 ± 0.5^1,2^	^1^2.962 ± 1.995^2^	1.5 ± 0.3	1.1517	^2^5.6 ± 0.6^2^
			Uninjured	7.1 ± 0.5^1^	^2^2.667 ± 0.615^2^	1.3 ± 0.3^1^	1.0945	^2^5.8 ± 0.6^3^
		3 days	Total	7.0 ± 0.1^1^	^1^0.015 ± 2.213^1^	7.0 ± 4.4	0.2320	^1^0.0 ± 4.4^1^
			Uninjured	7.0 ± 0.2^1^	^1^0.117 ± 0.103^1^	6.1 ± 0.5^3^	0.5028	^1^0.9 ± 0.5^1^
		10 days	Total	7.1 ± 0.1^1,2^	^1^0.086 ± 0.011^1^	–	0.3008	≈^1^2.0 ± 0.1^1,2^
			Uninjured	7.1 ± 0.1^1^	^1^0.172 ± 0.041^1^	4.9 ± 0.5^2^	0.4492	^1^2.2 ± 0.5^2^
		30 days	Total	7.5 ± 0.1^2^	^1^0.272 ± 0.104^1,2^	6.8 ± 0.1	0.2136	^1^0.7 ± 0.1^1^
			Uninjured	7.5 ± 0.2^1^	^1^0.434 ± 0.296^1^	6.6 ± 0.1^3^	0.4272	^1^0.9 ± 0.2^1^
	Biofilms	0 days	Total	6.2 ± 0.1^1,2^	^1^1.403 ± 0.160^1^	2.6 ± 0.1^1^	0.5000	^1^3.6 ± 0.1^3^
			Uninjured	5.9 ± 0.1^2,3^	^1^1.579 ± 0.174^1^	2.0 ± 0.2^1^	0.5330	^1^3.9 ± 0.2^2^
		3 days	Total	6.5 ± 0.2^2^	^1^1.500 ± 1.744^1^	4.4 ± 0.1^4^	0.4633	^1^2.1 ± 0.2^1,2^
			Uninjured	6.2 ± 0.3^3^	^2^1.324 ± 0.350^1^	3.5 ± 0.1^3^	0.5145	^2^2.7 ± 0.3^1^
		10 days	Total	5.9 ± 0.2^1^	^2^0.815 ± 0.231^1^	4.1 ± 0.1^3^	0.4524	^1^1.8 ± 0.2^1^
			Uninjured	5.7 ± 0.3^2^	^1^1.523 ± 1.150^1^	3.3 ± 0.2^2,3^	0.5394	^1^2.4 ± 0.4^1^
		30 days	Total	6.2 ± 0.2^1,2^	^2^0.774 ± 0.162^1^	3.9 ± 0.1^2^	0.4315	^2^2.3 ± 0.2^2^
			Uninjured	5.2 ± 0.3^1^	^1^0.739 ± 0.185^1^	3.0 ± 0.2^2^	0.5150	^2^2.2 ± 0.4^1^

*Inactivation parameters are determined for both the total and the uninjured population. ^1^Effect cell mode: for each microorganism, PAL age and population type, parameters of the Geeraerd et al. (2000) model bearing different superscripts (no numbers in common) are significantly different (P ≤ 0.05). ^2^Effect PAL age: for each microorganism, cell mode population type, parameters of the Geeraerd et al. (2000) model bearing different superscripts (no number in common) are significantly different (P ≤ 0.05).*

More specifically, when focusing on planktonic cells (both microorganisms), a satisfactory result could only be obtained with treatment directly following the activation of the liquids (PAL age = 0 days). This was indicated by a rapid decrease in cell level, followed by a long tailing phase when PAL treatment time increased. These findings were confirmed by the inactivation parameters, with *k_max_* and the *log-reduction* significantly higher at the PAL age of 0 days. In accordance, *N_res_* for *S.* Typhimurium planktonic cells increased with PAL storage time.

For biofilm inactivation, the solutions tended to become less effective with increasing age. However, they retained more potential compared to the PAL treatment of planktonic cells. With increasing PAL age, a (less significant) decrease of *k*_max_ and *log-reduction*, together with a significant increase of *N*_res_ could again be deducted for both microorganisms.

### PAL and Sublethal Injury

**FIGURE 5:**
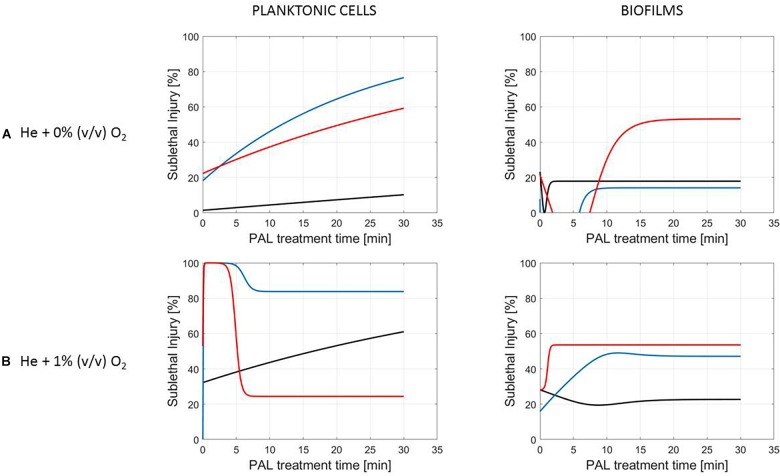
Evolution with time of the sublethal injury (%) of *L. monocytogenes* planktonic cells and biofilms toward the plasma activated liquid treatment time. Plasma activated liquids were created using two different gas compositions **(A)** He + 0% (v/v) O_2_ or **(B)** He + 1% (v/v) O_2_, and three different generation times (10, 20, and 30 min).

**FIGURE 6:**
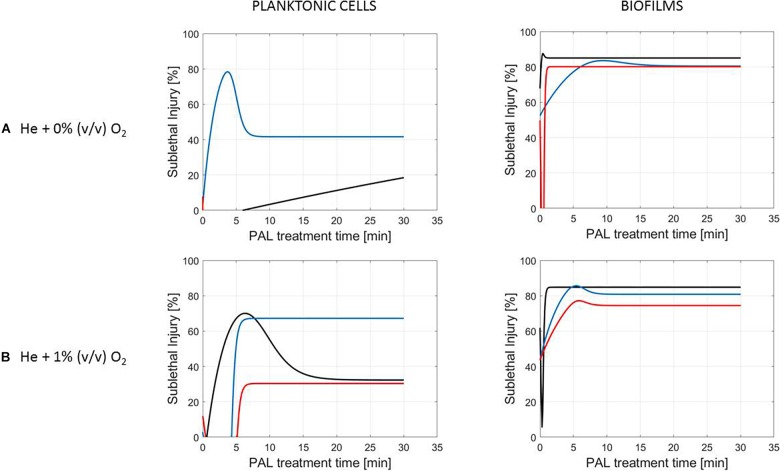
Evolution with time of the sublethal injury (%) of *S.* Typhimurium planktonic cells and biofilms toward the plasma activated liquid treatment time. Plasma activated liquids were created using two different gas compositions **(A)** He + 0% (v/v) O_2_ or **(B)** He + 1% (v/v) O_2_, and three different generation times (10, 20, and 30 min).

**FIGURE 7:**
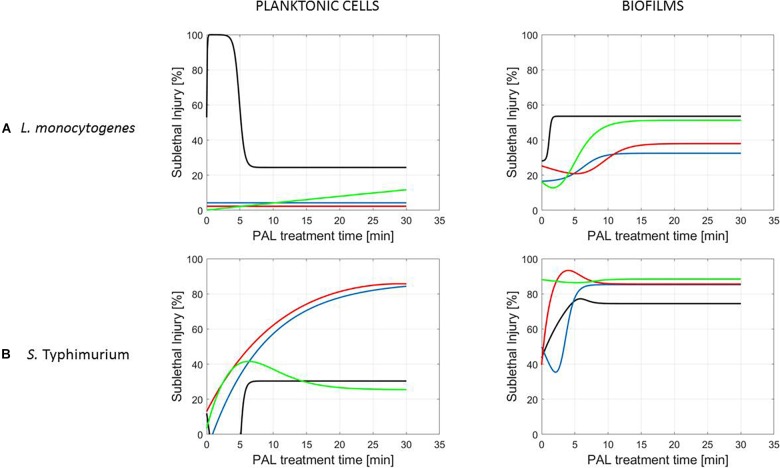
Evolution with time of the sublethal injury (%) of **(A)** *L. monocytogenes* or **(B)** *S.* Typhimurium planktonic cells and biofilms toward the plasma activated liquid treatment time using solutions of different ages (0, 3, 10, and 30 days).

Figure 5 (*L. monocytogenes*) and Figure 6 (*S.* Typhimurium) illustrate the percentage of sublethal injury (SI) as a function of the treatment time for the different cell modes, gas compositions and generation times, while Figure 7 presents SI for the four PAL ages (both microorganisms). In general, the range of SI percentages was very broad, and for some specific experimental conditions values around 80–100% SI were detected.

A clear accordance with the inactivation kinetics could be observed, as there a log-linear inactivation was often followed by a tail (Figure 2–4). Regarding sublethal injury, this was now represented by a fast increase in SI up to a maximum percentage, which was either (i) followed by a plateau with constant SI level or (ii) a small decrease in SI, again followed by this constant level.

In case no evident tail was present when assessing the inactivation kinetics (only log-linear inactivation), neither a plateau could be observed as the percentage of SI was still increasing [e.g., treatment of planktonic cells of *L. monocytogenes* with He + 0% (v/v) O_2_, Figure 5].

Finally, for experimental conditions where the inactivation efficacy proved limited (e.g., treatment of planktonic cells of *L. monocytogenes* at the PAL ages of 3, 10, and 30 days, Figure 7), also SI values remained low.

Although different factors (i.e., the microbial species and cell mode, PAL generation, and treatment conditions) had a definite impact on the inactivation efficacy, for most of them a specific trend on the SI evolution could not be detected. One of the observations made was that the percentage of SI tends to be higher for the PAL treatment of *S.* Typhimurium as compared to *L. monocytogenes*, which was most expressed when treating biofilms or for older PAL solutions.

## Discussion

### PAL Characteristics

The pH of the PAL solutions was most of all influenced by the CAP operating gas, and decreased in the presence of oxygen (Table 1). In literature, this pH drop was explained by the nitric acid formation through interaction of RNS, reactive oxygen species (ROS), and hydrolysis of water, that give rise to protons and hydroxyl radical (Ercan et al., 2013).

Hydrogen peroxide was abundantly detected in the PAL solutions (Table 1). Similar to literature, it was observed that its concentration (significantly) increased with generation time (Chauvin et al., 2017). In addition, the H_2_O_2_ concentration was expected to increase with the presence of oxygen in the CAP operating gas. Due to the higher probability of producing the OH radical from the generated atomic oxygen (electron induced dissociation), higher concentrations of hydrogen peroxide in the liquid were previously reported in presence of oxygen (Takamatsu et al., 2014; Ma et al., 2015). Due to high standard errors, no significant differences were observed (Table 1), but the values themselves confirmed this trend. In remark, the detected H_2_O_2_ concentrations were low compared to literature that aims at microbial inactivation by means of hydrogen peroxide, hinting PAL inactivation relies on more than the effect due to H_2_O_2_ present. For example, Stewart et al. (2000) used 50000 μM H_2_O_2_ (1 h treatment) and observed no inactivation of *Pseudomonas aeruginosa* biofilms.

Finally, both nitrite and nitrate were monitored in the PAL solutions (Table 1). NO, abundantly present in the plasma region above the water, can be easily oxidized to NO_2_. These radicals can consequently produce NO_2_^−^ and NO_3_^−^ in the water (Lu et al., 2017). At low pH, the nitrous acid also formed is not stable, and decomposes rapidly into nitrogen dioxide. If this nitrogen dioxide reacts with hydroxyl radicals, peroxynitrous acid is formed subsequently converting into nitrate. In addition, if NO_2_^−^ reacts with H_2_O_2_ under acidic conditions, nitrate can also form (Lukes et al., 2014; Chauvin et al., 2017).

As it was indicated that the different influencing factors (i.e., especially the gas composition and generation time) had an impact on the chemical composition of the PAL solutions, it can be reasoned beforehand that they will also have an important effect on the PAL inactivation efficacy.

### Influence of Microbial Species and Type of Cells

Experiments were conducted using either *L. monocytogenes* or *S.* Typhimurium, of which *L. monocytogenes* proved the most resistant against PAL treatment (Figure 2, 3 and Tables 2, 3). Similar to what was often observed regarding CAP treatment, a divergence in cell morphology between *L. monocytogenes* (Gram-positive) and *S.* Typhimurium (Gram-negative) can explain the different behavior between both microbial species when treated with PAL (Lee et al., 2006; Ermolaeva et al., 2011; Smet et al., 2017).

The results also indicated that the cell type (together with other factors) influenced the PAL inactivation (Figure 2, 3 and Tables 2, 3). In most of the observations above, the biofilms were more resistant to PAL as compared to planktonic cells (mainly for longer generation PAL times). This has previously been reported by Kamgang-Youbi et al. (2008), where PAL treatments were less efficient on adherent cells compared to planktonic cells (for both Gram-positive and Gram-negative bacteria). This can be explained by the fact that biofilms, significantly different from planktonic cells in behavior and physiology, are known to be highly tolerant to various stresses and antimicrobials, mostly due to the limited penetration of the EPS matrix, formation of persister cells, and quorum sensing-controlled protective mechanisms (Simoes et al., 2010; Gilmore et al., 2018).

In remark, for (i) 10 min PAL generation times and (ii) 0% (v/v) O_2_, PAL solutions were expected to have a lower efficacy based on the characterization (see section “PAL Characteristics”). However, higher inactivation efficiencies were observed for the biofilms compared to the planktonic cells for these conditions. The discrepancies observed can be explained due to a (partly) removal of the biofilms by washing out during rinsing (see protocol section “PAL Inactivation and Microbial Analysis”). In addition to the (limited) PAL inactivation itself, this phenomenon could explain the higher inactivation efficiency of biofilms compared to planktonic cells (Rabinovitch and Stewart, 2006).

In general, findings regarding the cell mode demonstrated that the inactivation efficacy of *L. monocytogenes* and *S.* Typhimurium planktonic cells and biofilms were directly proportional to the CAP factors used for PAL generation, as will be discussed in the following section.

### Influence of CAP Factors Used for PAL Generation

Regarding the generation of CAP, the addition of small amounts of oxygen will facilitate the creation of a higher amount of ROS (Laroussi and Leipold, 2004). It was therefore expected that varying the gas composition for the generation of PAL, would thus also influence the composition and the inactivation potential of the activated solutions (Figure 2, 3 and Tables 2, 3). The addition of oxygen during PAL generation resulted in additional reactive species which were transferred to the liquid and thus were involved in the inactivation process, explaining the higher inactivation efficacy observed. It is known that hydrogen peroxide, nitrites, and nitrates are the main stable long-lived species in PAL, facilitating microbial inactivation (Lu et al., 2017). In addition, Dezest et al. (2017) mentioned that short-lived species such as singlet oxygen, atomic oxygen or peroxynitrite may be involved. As can be expected, some important species are only transported into the liquid if O_2_ is present in the feed gas (Gorbanev et al., 2016). In general, oxidative stress induces membrane lipid peroxidation and the disruption of the cell membrane causing cytosolic leakage, which eventually leads to the cell death (Joshi et al., 2011; Ma et al., 2015; Tian et al., 2015).

The increasing PAL efficacy with longer generation times observed (Figure 2, 3 and Tables 2, 3) was in line with literature, as also Ma et al. (2015) found that prolonged generation times (5–15 min) increased the inactivation efficiency from 1.7 to 2.3 log-reduction of *S. aureus* planktonic cells.

### Influence of PAL Factors

The tail observed for the inactivation kinetics (Figure 2, 3) indicated that from that specific treatment time on, no additional microbial reduction could be obtained. This can be due to a difference in resistance of the cells within the population toward the treatment (Cerf, 1977). Moreover, if the population is assumed homogeneous, the tail can be explained by the fact that some cells (i) have adapted to the PAL treatment or (ii) are inaccessible to the treatment (e.g., for the biofilms) (Cerf, 1977). In addition, specific for inactivation by PAL, the tailing phase could also be hypothesized to appear as short-lived species only contribute to the inactivation up to a specific (short) treatment time. Especially expressed for planktonic cells, these findings proved that short-lived species also considerably attribute to the microbial inactivation mechanism (Ma et al., 2015).

Focusing on the cell mode, for stored PAL solutions higher inactivation efficacies were obtained for the biofilms. This could indicate that for PAL solutions with a lower microbial efficacy (i.e., due to PAL age), the partial removal of the biofilm still ensured a reduction in cell density (see section “Influence of Microbial Species and Type of Cells”). However, regardless the type of microorganism or cell mode, an increase in PAL age reduced its microbial inactivation efficacy (Figure 4 and Table 4). The observed resistance after storage, either expressed by a limited inactivation (planktonic cells) or tail (biofilms), hints at a loss of activity of the different species present within the PAL solutions. As during storage, the concentrations of the important long-lived species remained constant (see section “PAL Characteristics”), these findings could again indicate the importance of short-lived species within PAL solutions. Moreover, these results proved that storage conditions (e.g., lower temperatures) are extremely important (Shen et al., 2016).

### PAL and Sublethal Injury

Regarding sublethal injury as a function of treatment time (Figure 5–7), similarities with the inactivation behavior were observed. Often, like in Smet et al. (2017), the PAL treatment time at which the constant SI value is reached, overall coincides with the transition into the tailing phase. If no tail was present for the inactivation kinetics, nor a plateau was reached for the SI, this corresponds to the experimental conditions where further inactivation might still be obtained if PAL treatment times would be extended (Smet et al., 2017).

## Conclusion

Plasma activated liquids can be assumed as an alternative for CAP, as this research illustrated its antimicrobial efficacy against foodborne pathogens. In this study, the different influencing factors, related to (i) the microbial species and type of cells, (ii) the PAL generation, and (iii) PAL treatment, prove to significantly affect the inactivation potential. Chemical characterization of the PAL solutions indicated the presence of long-lived species (mainly hydrogen peroxide and nitrate) and acidification of the liquid, both known to contribute to bacterial inactivation. First of all it was illustrated that apart from the microbial species, mainly the cell mode (planktonic cells vs. biofilms) influences the PAL treatment, with biofilms often exhibiting a higher resistance toward the treatment. Regarding its generation, higher inactivation efficacies were observed with the addition of oxygen in the CAP operating gas and for longer generation times. As confirmed by the chemical characterization of the PAL solutions, higher amounts of long-lived species are measured by (i) using He + 1% (v/v) O_2_ and (ii) extending generation times to 30 min. Regarding treatment times, the appearance of tailing within the kinetics might indicate the additional importance of short-lived species for microbial inactivation. Finally, loss of PAL activity during subsequent storage illustrated a need for further research (e.g., at different storage temperatures) to improve the potential of PAL for applications ensuring food safety.

## Data Availability

The datasets generated for this study are available on request to the corresponding author.

## Author Contributions

CS, MG, and JI conceived and supervised the study. CS and MG carried out the methodology and data validation, conducted the formal analysis, and wrote the original draft of the manuscript. CS carried out the software and project administration, visualized the study, and responsible for data curation. CS, AK, and ME investigated the study. JW and JI were responsible for the resources. CS, MG, JW, and JI wrote, reviewed, and edited the manuscript. JI was involved in funding acquisition.

## Conflict of Interest Statement

The authors declare that the research was conducted in the absence of any commercial or financial relationships that could be construed as a potential conflict of interest.

## References

[B1] Aharoni Y. Fallik E. Copel A. Gil M. Grinberg S. Klein J. D. (1997). Sodium bicarbonate reduces postharvest decay development on melons. *Postharvest Biol. Technol.* 10 201–206. 10.1016/s0925-5214(97)01412-9

[B2] Belgian Co-ordinated Collections of Micro-organisms [BCCM] (2017). *Bacterial Cultures.* Available at: http://bccm.belspo.be/catalogues/lmg-catalogue-search (accessed January 18, 2019).

[B3] Busch S. V. Donnelly C. W. (1992). Development of a repair-enrichment broth for resuscitation of heat-injured *Listeria monocytogenes* and *Listeria innocua*. *Appl. Environ. Microbiol.* 58 14–20. 1531746 10.1128/aem.58.1.14-20.1992 PMC195165

[B4] Cerf O. (1977). A review. Tailing of survival curves of bacterial spores. *J. Appl. Bacteriol.* 42 1–19. 10.1111/j.1365-2672.1977.tb00665.x 323208

[B5] Chauvin J. Judée F. Yousfi M. Patricia Vicendo P. Merbahi N. (2017). Analysis of reactive oxygen and nitrogen species generated in three liquid media by low temperature helium plasma jet. *Sci. Rep.* 7:4562. 10.1038/s41598-017-04650-4 28676723 PMC5496897

[B6] Deng X. Shi J. Kong M. (2006). Physical mechanisms of inactivation of *Bacillus subtilis* spores using cold atmospheric plasmas. *IEEE Trans. Plasma Sci.* 34 1310–1316. 10.1128/AEM.00583-12 22582068 PMC3416436

[B7] Dezest M. Bulteau A. Quinton D. Chavatte L. Le Bechec M. Cambus J. P. (2017). Oxidative modification and electrochemical inactivation of *Escherichia coli* upon cold atmospheric pressure plasma exposure. *PLoS One* 12:e0173618. 10.1371/journal.pone.0173618 28358809 PMC5373509

[B8] Ercan U. K. Wang H. Ji H. Fridman G. Brooks A. D. Joshi S. G. (2013). Non equilibrium plasma-activated antimicrobial solutions are broad-spectrum and retain their efficacies for extended period of time. *Plasma Process. Polym.* 10 544–555. 10.1002/ppap.201200104

[B9] Ermolaeva S. A. Varfolomeev A. F. Chernukha M. Y. Yurov D. S. Vasiliev M. M. Kaminskaya A. A. (2011). Bactericidal effects of non-thermal argon plasma in vitro, in biofilms and in the animal model of infected wounds. *J. Med. Microbiol.* 60 75–83. 10.1099/jmm.0.020263-0 20829396

[B10] Geeraerd A. Herremans C. Van Impe J. F. (2000). Structural model requirements to describe microbial inactivation during a mild heat treatment. *Int. J. Food Microbiol.* 59 185–209. 10.1016/s0168-1605(00)00362-7 11020040

[B11] Giaouris E. Heir E. Hébraud M. Chorianopoulos N. Langsrud S. Moretro T. (2014). Attachment and biofilm formation by foodborne bacteria in meat processing environments: causes, implications, role of bacterial interactions and control by alternative novel methods. *Meat Sci.* 97 298–309. 10.1016/j.meatsci.2013.05.023 23747091

[B12] Gilmore B. F. Flynn P. B. O’Brien S. Hickok N. Freeman T. Bourke P. (2018). Cold plasmas for biofilm control: opportunities and challenges. *Trends Biotechnol.* 36 627–638. 10.1016/j.tibtech.2018.03.007 29729997 PMC12168448

[B13] Gómez-López V. M. (2012). *Decontamination of Fresh and Minimally Processed Produce*, 1st Edn. Hoboken, NJ: John Wiley & Sons, Inc.

[B14] Goodburn C. Wallace C. (2013). The microbiological efficacy of decontamination methodologies for fresh produce: a review. *Food Control* 32 418–427. 10.1016/j.foodcont.2012.12.012

[B15] Gorbanev Y. O’Connell D. Chechik V. (2016). Non-thermal plasma in contact with water: the origin of species. *Chemistry* 22 3496–3505. 10.1002/chem.201503771 26833560 PMC4797710

[B16] Govaert M. Smet C. Baka M. Janssens T. Van Impe J. (2018a). Influence of incubation conditions on the formation of model biofilms by *Listeria monocytogenes* and *Salmonella Typhimurium* on abiotic surfaces. *J. Appl. Microbiol.* 125 1890–1900. 10.1111/jam.14071 30117654

[B17] Govaert M. Smet C. Vergauwen L. Ecimovic B. Walsh J. L. Baka M. (2018b). Influence of plasma characteristics on the efficacy of cold atmospheric plasma (CAP) for inactivation of *Listeria monocytogenes* and *Salmonella Typhimurium* biofilms. *Innov. Food Sci. Emerg.* 52 376–386. 10.1016/j.ifset.2019.01.013

[B18] Hassan A. Usman J. Kaleem F. Omair M. Khalid A. Iqbal M. (2011). Evaluation of different detection methods of biofilm formation in the clinical isolates. *Braz. J. Infect. Dis.* 15 305–311. 10.1590/s1413-86702011000400002 21860999

[B19] Jablonowski H. von Woedtke T. (2015). Research on plasma medicine-relevant plasma–liquid interaction: what happened in the past five years? *Clin. Plasma Med.* 3 42–52. 10.1016/j.cpme.2015.11.003 25743772

[B20] Joshi S. G. Cooper M. Yost A. Paff M. Ercan U. K. Fridman G. (2011). Nonthermal dielectric-barrier discharge plasma-induced inactivation involves oxidative DNA damage and membrane lipid peroxidation in *Escherichia coli*. *Antimicrob. Agents Chemother.* 55 1053–1062. 10.1128/AAC.01002-10 21199923 PMC3067084

[B21] Kamgang-Youbi G. Herry J. M. Brisset J. L. Bellon-Fontaine M.-N. Doubla A. Naïtali M. (2008). Impact on disinfection efficiency of cell load and of planktonic/adherent/detached state: case of *Hafnia alvei* inactivation by plasma activated water. *Appl. Microbiol. Biotechnol.* 81 449–457. 10.1007/s00253-008-1641-9 18769918

[B22] Kelly-Wintenberg K. Hodge A. Montie T. C. Deleanu L. Sherman D. Reece Roth J. (1999). Use of a one atmosphere uniform glow discharge plasma to kill a broad spectrum of microorganisms. *J. Vac. Sci. Technol. A* 17 1539–1544. 10.1116/1.581849

[B23] Kim S. Wei C. (2012). “Biofilms,” in *Decontamination of Fresh and Minimally Processed Produce*, 1st Edn, ed. Gómez-López V. M. (Hoboken, NJ: John Wiley & Sons, Inc.), 59–76.

[B24] Korachi M. Gurol C. Aslan N. (2010). Atmospheric plasma discharge sterilization effects on whole cell fatty acid profiles of *Escherichia coli* and *Staphylococcus aureus*. *J. Electrostat.* 68 508–512. 10.1016/j.elstat.2010.06.014

[B25] Kumar G. C. Anand S. K. (1998). Significance of microbial biofilms in food industry: a review. *Int. J. Food Microbiol.* 42 9–27. 10.1016/s0168-1605(98)00060-9 9706794

[B26] Laroussi M. Alexeff I. Kang W. L. (2000). Biological decontamination by nonthermal plasmas. *IEEE Trans. Plasma Sci.* 28 184–188. 10.1109/27.842899

[B27] Laroussi M. Leipold F. (2004). Evaluation of the roles of reactive species, heat, and UV radiation in the inactivation of bacterial cells by air plasmas at atmospheric pressure. *Int. J. Mass Spectrom.* 233 81–86. 10.1016/j.ijms.2003.11.016

[B28] Lee K. Paek K. Ju W. T. Lee Y. (2006). Sterilization of bacteria, yeast and bacterial endospores by atmospheric-pressure cold plasma using helium and oxygen. *J. Microbiol.* 44 269–275. 16820756

[B29] Lopez-Galvez F. Ragaert P. Palermo L. A. Eriksson M. Devlieghere F. (2013). Effect of new sanitizing formulations on quality of fresh-cut iceberg lettuce. *Postharvest Biol. Technol.* 85 102–108. 10.1016/j.postharvbio.2013.05.005

[B30] Lu P. Boehm D. Bourke P. Cullen P. J. (2017). Achieving reactive species specificity within plasma-activated water through selective generation using air spark and glow discharges. *Plasma Process. Polym.* 14:e1600207 10.1002/ppap.201600207

[B31] Lukes P. Dolezalova E. Sisrova I. Clupek M. (2014). Aqueous-phase chemistry and bactericidal effects from an air discharge plasma in contact with water: evidence for the formation of peroxynitrite through a pseudo-second-order post-discharge reaction of H2 O2 and HNO2. *Plasma Sources Sci. Technol.* 23:015019 10.1088/0963-0252/23/1/015019

[B32] Ma R. Wang G. Tian Y. Wang K. Zhang J. Fang J. (2015). Non-thermal plasma-activated water inactivation of food-borne pathogen on fresh produce. *J. Hazard. Mater.* 300 643–651. 10.1016/j.jhazmat.2015.07.061 26282219

[B33] Miles A. A. Misra S. S. Irwin J. O. (1938). The estimation of the bactericidal power of the blood. *J. Hyg.* 38 732–749. 10.1017/s002217240001158x 20475467 PMC2199673

[B34] Misra N. N. Schluter O. Cullen P. J. (2016). “Plasma in food and agriculture,” in *Cold Plasma in Food and Agriculture*, eds Misra N. N. Schluter O. K. Cullen P. J. (Cambridge, MA: Academic Press), 1–16. 10.1016/b978-0-12-801365-6.00001-9

[B35] Misra N. N. Tiwari B. K. Raghavarao K. S. M. S. Cullen P. J. (2011). Nonthermal plasma inactivation of food-borne pathogens. *Food Eng. Rev.* 3 159–170. 10.1111/lam.13095 30412302

[B36] Moisan M. Barbeau J. Moreau S. Pelletier J. Tabrizian M. Yahia L. (2001). Low-temperature sterilization using gas plasmas: a review of the experiments and an analysis of the inactivation mechanisms. *Int. J. Pharm.* 226 1–21. 10.1016/s0378-5173(01)00752-9 11532565

[B37] Rabinovitch C. Stewart P. S. (2006). Removal and inactivation of *Staphylococcus epidermidis* biofilms by electrolysis. *Appl. Environ. Microbiol.* 72 6364–6366. 10.1128/aem.00442-06 16957263 PMC1563645

[B38] Rico D. Martin-Diana A. B. Barat J. M. Barry-Ryan C. (2007). Extending and measuring the quality of fresh-cut fruit and vegetables: a review. *Trends Food Sci. Technol.* 18 373–386. 10.1016/j.tifs.2007.03.011

[B39] Shen J. Tian Y. Li Y. Ma R. Zhang Q. Zhang J. (2016). Bactericidal effects against *S. aureus* and physicochemical properties of plasma activated water stored at different temperatures. *Sci. Rep.* 6:28505. 10.1038/srep28505 27346695 PMC4921907

[B40] Simoes M. Simoes L. C. Vieira M. J. (2010). A review of current and emergent biofilm control strategies. *LWT-Food Sci. Technol.* 43 573–583. 10.3390/molecules21070877 27399652 PMC6274140

[B41] Smet C. Noriega E. Rosier F. Walsh J. L. Valdramidis V. P. Van Impe J. F. (2017). Impact of food model (micro)structure on the microbial inactivation efficacy of cold atmospheric plasma. *Int. J. Food Microbiol.* 240 47–56. 10.1016/j.ijfoodmicro.2016.07.024 27507138

[B42] Stewart P. S. Roe F. Rayner J. Elkins J. G. Lewandowski Z. Ochsner U. A. (2000). Effect of catalase on hydrogen peroxide penetration into *Pseudomonas aeruginosa* Biofilms. *Appl. Environ. Microbiol.* 66 836–838. 10.1128/aem.66.2.836-838.2000 10653761 PMC91906

[B43] Sun P. Wu H. Bai N. Zhou H. Wang R. Feng H. (2012). Inactivation of *Bacillus subtilis* spores in water by a direct-current, cold atmospheric-pressure air plasma microjet. *Plasma Process. Polym.* 9 157–164. 10.1002/ppap.201100041

[B44] Takamatsu T. Uehara K. Sasaki Y. Miyahara H. Matsumura Y. Iwasawa A. (2014). Investigation of reactive species using various gas plasmas. *RSC Adv.* 4 39901–39905. 10.1039/c4ra05936k

[B45] Thirumdas R. Kothakota A. Annapure U. Siliveru K. Blundell R. Gatt R. (2018). Plasma activated water (PAW): chemistry, physico-chemical properties, applications in food and agriculture. *Trends Food Sci. Technol.* 77 21–31. 10.1016/j.tifs.2018.05.007

[B46] Tian Y. Ma R. Zhang Q. Feng H. Liang Y. Zhang J. (2015). Assessment of the physicochemical properties and biological effects of water activated by non-thermal plasma above and beneath the water surface. *Plasma Process. Polym.* 12 439–449. 10.1002/ppap.201400082

[B47] Ziuzina D. Han L. Cullen P. J. Bourke P. (2015). Cold plasma inactivation of internalised bacteria and biofilms for *Salmonella enterica* serovar Typhimurium, *Listeria monocytogenes* and *Escherichia coli*. *Int. J. Food Microbiol.* 210 53–61. 10.1016/j.ijfoodmicro.2015.05.019 26093991

